# Comparative Analysis of *Akebia trifoliata* Fruit Softening at Different Flesh Ripening Stages Using Tandem Mass Tag Technology

**DOI:** 10.3389/fnut.2021.684271

**Published:** 2021-07-05

**Authors:** Juan Niu, Zhimin Sun, Yaliang Shi, Kunyong Huang, Yicheng Zhong, Jing Chen, Jianhua Chen, Mingbao Luan

**Affiliations:** Institute of Bast Fiber Crops, Chinese Academy of Agricultural Sciences, Ministry of Agriculture, Changsha, China

**Keywords:** fruit softening, gene expression, *Akebia trifoliata*, tandem mass tag, different flesh stages

## Abstract

Owing to its medicinal and high nutritional values, *Akebia trifoliata* can be considered as a new type of medicinal and edible homologous resources, and it has begun to be widely cultivated in many areas of China. Over-softening of fruit would affect the sensorial quality, utilization rate, and consumer acceptance of the fruit postharvest. However, fruit softening has not been characterized and the molecular mechanism underlying *A. trifoliata* fruit softening during ripening remains unclear. A comparative proteomic analysis was performed on the fruit at three developmental stages using tandem mass tag technology. In total, 2,839 proteins and 302 differentially abundant proteins (DAPs) were identified. Bioinformatics analysis indicated that most DAPs were implicated in oxidoreductase activity, protein domain-specific binding and pyruvate metabolism. Moreover, 29 DAPs associated with cell wall metabolism, plant hormone, and stress and defense response pathways were validated using quantitative PCR. Notably, pectinesterase, pectate lyase, and β-galactosidase, which are involved in cell wall degradation, as well as gibberellin regulated protein, cysteine protease, thaumatin-like protein and heat shock proteins which is involved in plant hormone, and stress and defense response, were significantly up-regulated in softening fruit compared with the levels in non-softening fruit. This indicated that they might play key roles in *A. trifoliata* fruit softening. Our findings will provide new insights into potential genes influencing fruit softening traits of *A. trifoliata*, which will help to develop strategies to improve fruit quality and reduce softening-related losses.

## Introduction

*Akebia trifoliata* (Thunb.) Koidz, a wild perennial liana of the Lardizabalaceae family, is widely distributed in many provinces in China and has been widely used in traditional Chinese medicine for at least 2,000 years (1). *Akebia trifoliata* has been listed in the Chinese Pharmacopeia 2015 edition. The fruit of *A. trifoliata* has been prized for its diuretic, antiphlogistic, antioxidant, anti-bacterial, anti-inflammation, anti-tumor, blood pressure-lowering properties (2–4) and has a delicious sweet taste and can be considered a nutrient or health food supplement because it is rich in vitamins, minerals, crude proteins, saccharides, and amino acids required for human health (5, 6). Additionally, the flesh extract of *A. trifoliata* has strong tyrosinase inhibitory activity and has the potential to be used in the development of whitening health care products (7). Because of its high economic, nutritional, and medicinal values, *A. trifoliata* is a new type of medicinal and edible plant with high development potential.

However, the fruit undergoes flesh softening and pericarp de-greening after harvest; therefore, softening is a critical process to ensure postharvest quality (8). Over-softening is associated with easy rotting mediated by bacterial infection, reduced marketability, decreased disease resistance, reduced shelf life, and accelerated fruit senescence, affecting the utilization rate of the fruit, and causing significant losses in commercial value and yields (4, 9, 10). Therefore, despite the importance of *A. trifoliata* fruit, the fruit quality decreases rapidly after harvest owing to a limited shelf life of only 3–5 days at room temperature (25°C), thereby severely limiting its marketability and value.

Fruit softening is reportedly related to the degradation/modification of the cell wall (9, 11). Various proteins/genes involved in cell wall modifications in fruit softening during ripening have been studied in several fruit, such as the banana (9), apple (11), and strawberry (12). For example, polygalacturonase (PG), pectin esterase (PE), pectin methylesterase, and pectate lyase (PL) are considered the main hydrolases involved in pectin solubilization and depolymerization during fruit ripening (13, 14). Xyloglucan endo-transglucosylase/hydrolase (XTH) and expansin (EXP) are the main hydrolases involved in remodeling or modification of hemicellulose (15). Furthermore, α-arabinofuranosidase (ASD), β-galactosidase (β-GAL), and β-xylosidase (BXL) are involved in cell wall modifications during fruit ripening (16).

However, the process of fruit ripening is a complicated and the mechanism underlying this process in fleshy fruit remains unclear, which has hindered an understanding of fruit softening and the increase in the postharvest shelf life of the fruit. Moreover, only the expression patterns of several genes from transcriptome data have been investigated during fruit ripening (14, 17). The lack of genetic data has greatly limited extensive and intensive research on this fruit crop. Moreover, owing to post-transcriptional and post-translational regulation, the gene transcript levels detected do not always correspond well with the abundances of proteins that determine trait expression (18). Proteomics might be a promising approach to uncover the complex physiological processes associated with fruit ripening and softening at the global protein level, which play a crucial role in fruit ripening and physiological stress responses, such as those of pomegranate (19), apple (20), banana (21), and litchi (22). To explore the underlying mechanisms that determine fruit softening in *A. trifoliata*, a comparative proteomic analysis was performed with tandem mass tag (TMT) technology. The findings will provide a foundation for the molecular basis of fruit softening in harvested *A. trifoliata* fruit.

## Materials and Methods

### Plant Materials

**Figure 1:**
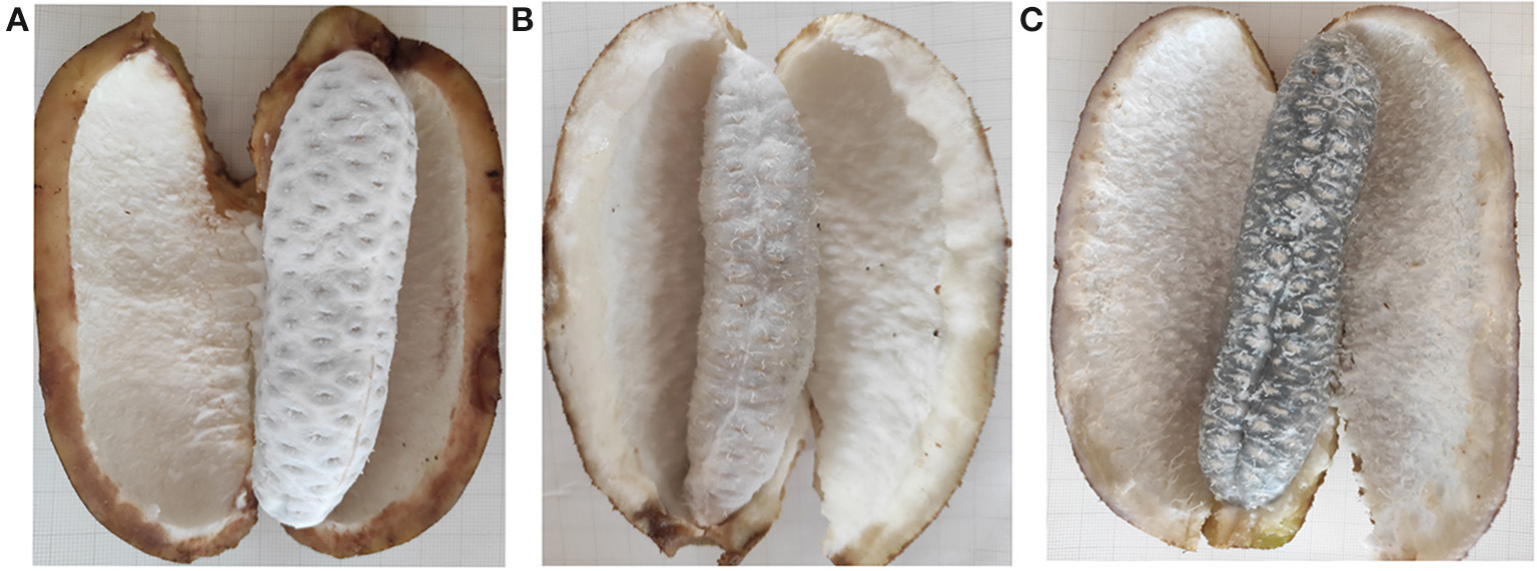
The morphology changes of “Nong No. 8” flesh at different development stages. **(A)** The morphology of the *A. trifoliata* flesh at unripe stage without fruit softening (UR). **(B)** The *A. trifoliata* flesh remains in hard status but the pericarp begins to crack longitudinally along with its ventral suture (HR). **(C)** The *A. trifoliata* flesh is fully matured, soft and edible, accompanied by complete cracking of the pericarp (FR).

*Akebia trifoliata* sample “Nong No. 8”, from a 9-year-old tree, which bloomed in early April and ripened in early October in Changsha, Hunan Province, was selected as the research material. From early April to mid-April, the average temperature is 24°C during the day, 15°C at night, 31°C/21°C maximum and 11°C/8 °C minimum. April rainfall days in Changsha are 11 days, with an average rainfall of 126 mm. The average temperature is 26°C/17°C, maximum 29°C/18°C and minimum 19°C/16°C in early October and an average rainfall of 72.3 mm in October. The pericarp of *A. trifoliata* cracks longitudinally along the ventral suture with fruit ripening (14). The fruit is hard, the flesh is white, and the pericarp ventral suture is closed when the fruit is immature (UR, Figure 1A). As the fruit begins to mature, the flesh is still hard, but the pericarp begins to soften and crack longitudinally along the ventral suture (HR, Figure 1B). When the fruit is fully mature, the flesh becomes glittering and translucent, soft, and edible, accompanied by completely cracking of the pericarp (FR, Figure 1C). The experimental material at three developmental stages were randomly collected every 10 days during fruit ripening (September 18, temperature: 32°C/24°C; September 28, temperature: 25°C/18°C; October 8, 2018, temperature: 26°C/18°C). Three fruit flesh were mixed into one biological replicate, and each sample consisted of three biological replicates, and the flesh was immediately frozen in liquid nitrogen for proteome studies and reverse transcription quantitative polymerase chain reaction (RT-qPCR).

### Protein Extraction

Total protein was extracted from *A. trifoliata* flesh using trichloroacetic acid (TCA)/acetone, as previously described (23). TCA/acetone was placed in the sample at −20°C for 4 h and then centrifuged at 4°C for 40 min at 20,000 × *g*. The supernatant was discarded, and the precipitate was washed three times using ice-cold acetone. The precipitate was freeze-dried and then re-suspended in buffer. The lysate was centrifuged for 40 min after sonication and boiled for 15 min. Lastly, the supernatant was filtered, and the filtrate was quantified using the BCA Protein Assay Kit (BioRad, Hercules, USA).

### Trypsin Digestion and TMT Labeling

The protein solution was incorporated into 100 mM dithiothreitol, and repeated ultrafiltration was performed with UA buffer (8 M urea, 150°C mM tris-HCl; pH 8.0). Iodoacetamide (100 mM IAA in UA buffer) was then added to the sample and incubated in the dark for 30 min at 2°C. The filtrate was washed three times with UA buffer, followed by triethylammonium bicarbonate (TEAB), and then digested with trypsin at a ratio of 40:1 (protein: trypsin) overnight in TEAB buffer at 37°C. The samples were labeled with TMTs, including 126-tag (UR), 127-tag (HR), and 128-tag (FR) (Thermo Fisher Scientific, Waltham, MA, USA), after desalting of trypsin peptides using an XC18 column (Phenomenex, Torrance, USA) and drying under a vacuum. The labeled peptide mixtures were combined at equal ratios and then fractionated using a high pH reversed-phase peptide fractionation kit (Thermo Scientific, Waltham, MA).

### Reverse-Phase Chromatography and Mass Spectrometry

The reverse-phase trap column (Acclaim PepMap 100 C18, 100 μm× 2 cm; Thermo Scientific) was used for fractionation of the TMT-labeled peptide mixture and then separated with an analytical 75-μm inner diameter C18 column (10 cm) in 0.1% formic acid (FA) buffer. The peptide mixture was eluted using gradient elution [84% acetonitrile in 0.1% FA] at a flow rate of 300 nL/min. The eluent was injected directly into a Q-Exactive mass spectrometer (Thermo Scientific, Waltham, MA, USA) connected with an Easy-nLC 1000 UPLC system (Thermo Scientific). Entire peptides were acquired at 70,000 resolutions with an automatic full MS scan of 300–1,800 m/z in the Orbitrap at an MS scan resolution of 17,500 with a fixed mass at m/z 200. The top 10 abundant precursor ions were selected with a dynamic exclusion duration of 40.0 s for higher-energy collision dissociation fragmentation at a normalized collision energy of 30%. Automatic gain control and maximum injection time were set as 3E6 and 10 ms, respectively.

### Database Search and Data Analyses

The MASCOT engine (Matrix Science, London, UK; version 2.2) embedded into Proteome Discoverer 1.4 software based on our previous transcriptome data was used to analyze the raw data files with the following settings: maximum missed cleavage, 2; fixed modifications, carbamidomethyl (C) and variable modification: oxidation (M); peptide mass tolerance, 0.1 Da; peptide ions, 20 ppm; peptide-spectrum matches and protein identification levels of FDR, ≤ 0.01; and unique peptides were employed for the protein identification and quantification. A fold-change of >1.2 or <0.83 with a *p* < 0.05 was set for differentially abundant proteins (DAPs). The functional identification of the identified proteins was performed using the GO database, and pathway enrichment analyses were performed with the Kyoto Encyclopedia of Genes and Genomes pathway (KEGG; www.genome.jp/kegg) databases. Heatmap 2.0 in the gplot R package and STRING 9.0 software (http://string-db.org) were used for clustering analysis and functional network analysis of the DAPs, respectively. The mass spectrometry proteomics data have been deposited in the iProX database (http://www.iprox.org; accession number: IPX0002000000; PXD number: PXD017282).

### RT-qPCR

The flesh and pericarp of “Nong No. 8” fruit at different development stages (UR, HR, and FR) were analyzed using RT-qPCR to evaluate the expression profiles of selected DAPs. Total RNA was extracted according to the method previously described by Niu et al. (14), and the cDNA synthesis was performed with the Hiscript qRT SuperMix (Vazyme, Biotech). The primers of an internal control gene (*EF-*α) and candidate genes were designed with Primer 5.0 (Supplementary Table 1), and the reaction was carried out using SYBR Green PCR master mix (Aidlab Biotechnologies, Co., Ltd) in a Bio-Rad CFX96 PCR system (Bio-Rad, Richmond, CA, USA). The data were analyzed using the Delta Ct method from three independent biological replicates with a *t*-test at *p* < 0.05 using GraphPad Prism 8 software.

## Results

### Quantitative Proteomics Analysis

**Figure 2:**
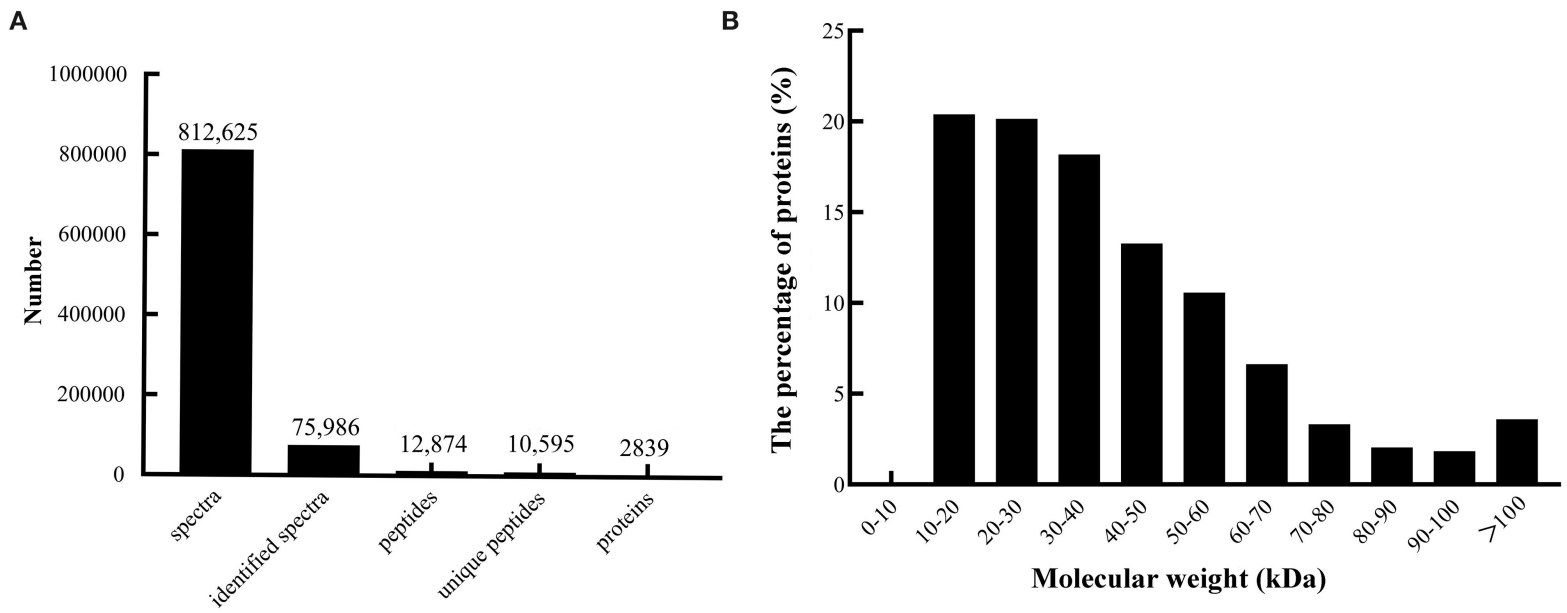
Summary of peptides and proteins that were identified from TMT proteomics by searching against the database. **(A)** Number of peptides that match proteins using MASCOT. **(B)** Distribution of the proteins that were identified among different molecular weights.

A quantitative proteomic analysis between unripe and ripe flesh was performed to understand the trait of fruit softening in *A. trifoliata*. The mass error distribution of identified peptides was near zero, and most of the errors were <0.02 Da, suggesting that the mass accuracy of the identified MS data conformed to the requirements. In total, 812,625 spectra (75,986 identified), 12,874 peptides (10,595 unique), and 2,839 proteins were identified (Figure 2A). The molecular weight distribution of the identified proteins was broad, ranging from 9 to 397 kDa, among which the highest distribution was 10–30 kDa (Figure 2B).

**Figure 3:**
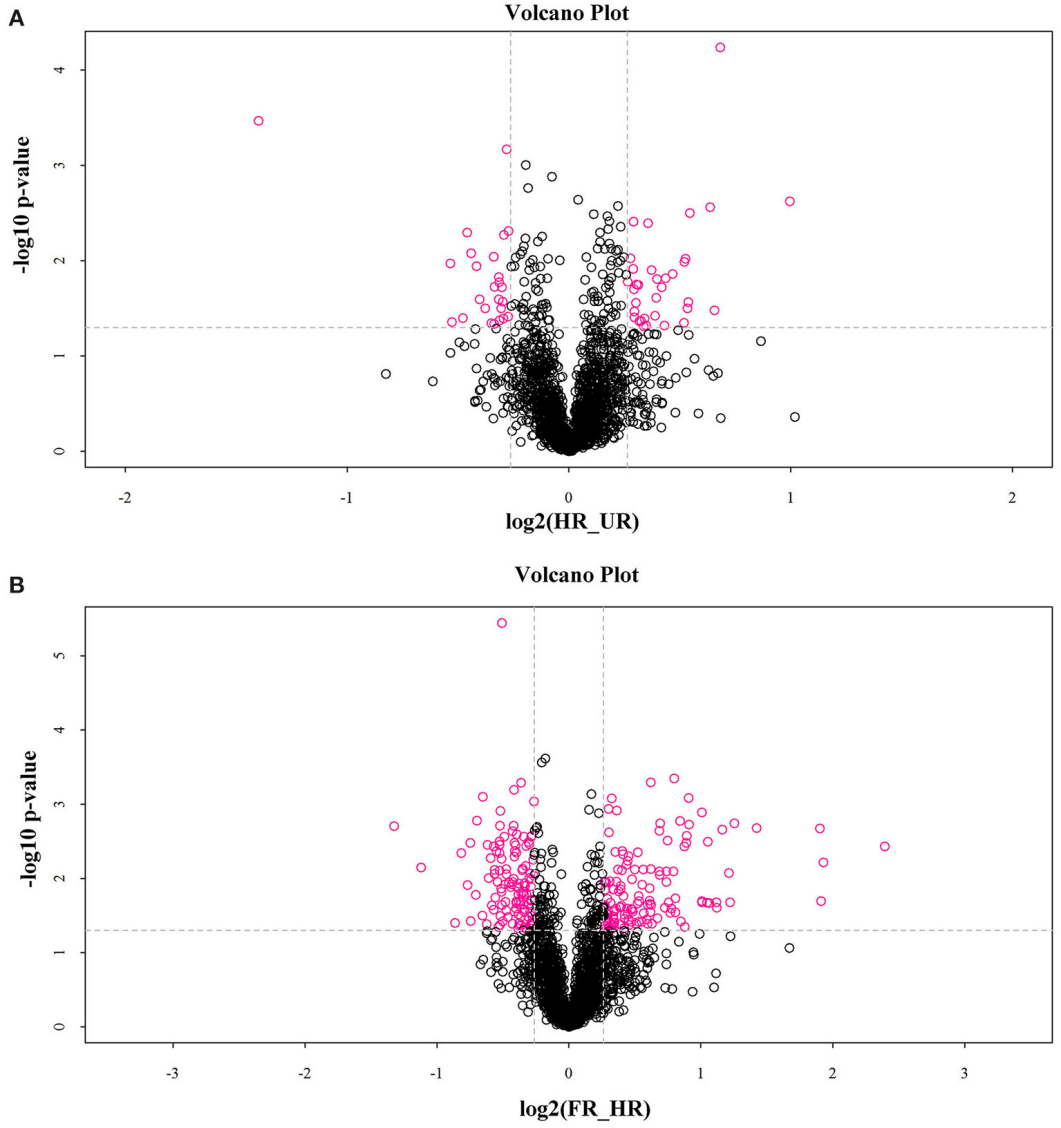
Volcano plot depicting the proteomics data of *A. trifoliata*. **(A)** Volcano plot depicting the proteomics data in HR_UR. **(B)** Volcano plot depicting the proteomics data in FR_HR. Absolute log10 and log2 fold changes are plotted on the y-axis and x-axis, respectively. Horizontal dotted line presents *p* values of 0.05 cut-off position while the vertical dotted lines discriminate between proteins having absolute log2 fold change of 1. Pink dots represent a log2 fold change >1 with *p* < 0.05 in protein expression. Black dots indicate no difference in protein expression.

In total, 302 *A. trifoliata* fruit ripening-related DAPs (155 up- and 133 down-regulated, and 14 co-expressed) were identified (Table 1), which are presented in the volcano plot (Figure 3). Among these DAPs, 27 were up- regulated and 18 down-regulated DAPs in beginning ripening fruit compared with immature fruit (HR_UR). There were 128 up-regulated and 115 down-regulated proteins in fully ripened fruit compared with those in the beginning ripening fruit (FR_ HR). Additionally, 14 DAPs were co-expressed in HR_UR (7 up- and 7 down-regulated) and FR_ HR (6 up- and 8 down-regulated; Supplementary Table 2).

**Table 1:** Summary of proteins detected from TMT sequence data.

	**All**	**HR_UR**	**FR_HR**
Total spectra	812,625		
Identified spectra	75,986		
Identified peptides	12,874		
unique peptides	10,595		
Unique proteins	2,839		
Significantly DAPs	302	45	243
Up-regulated	155	27	128
Down-regulated	133	18	115
Shared proteins	14	14	14
Shared proteins (up-regulated)		7	6
Shared proteins (down-regulated)		7	8

### Functional Classification of the Identified DAPs

**Figure 4:**
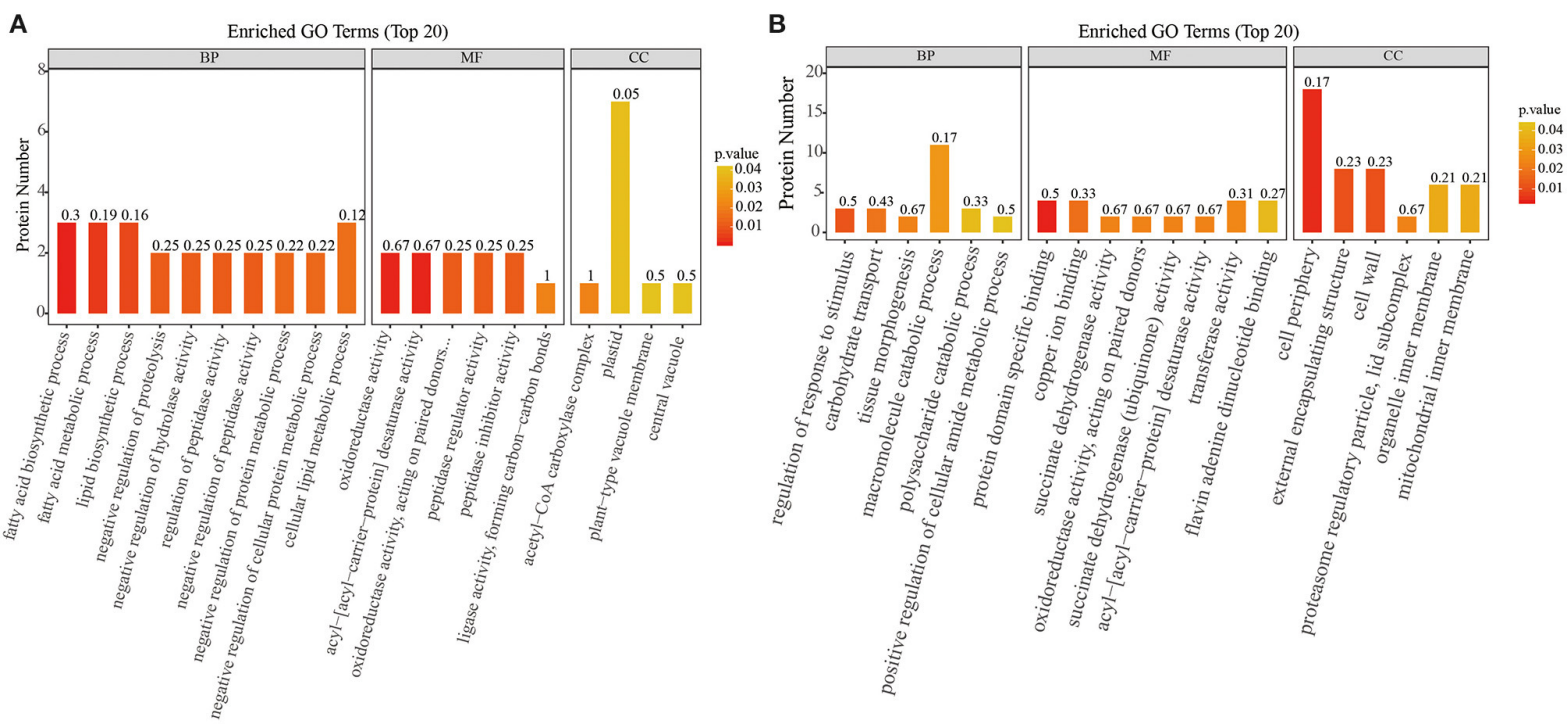
GO enrichment analysis of differentially abundant proteins (DAPs) in HR_UR and FR_HR group. **(A)** HR_UR; **(B)** FR_HR. The horizontal axis shows the top 20 enriched GO terms in the biological process, molecular function, and cellular component categories. The vertical axis shows DAPs enriched in each term. The height and color of each histogram indicates the number of proteins and *p* values of the enriched term. The label of each histogram indicates the rich factor, which represents the ratio of DAPs to total proteins identified in GO functional category.

**Figure 5:**
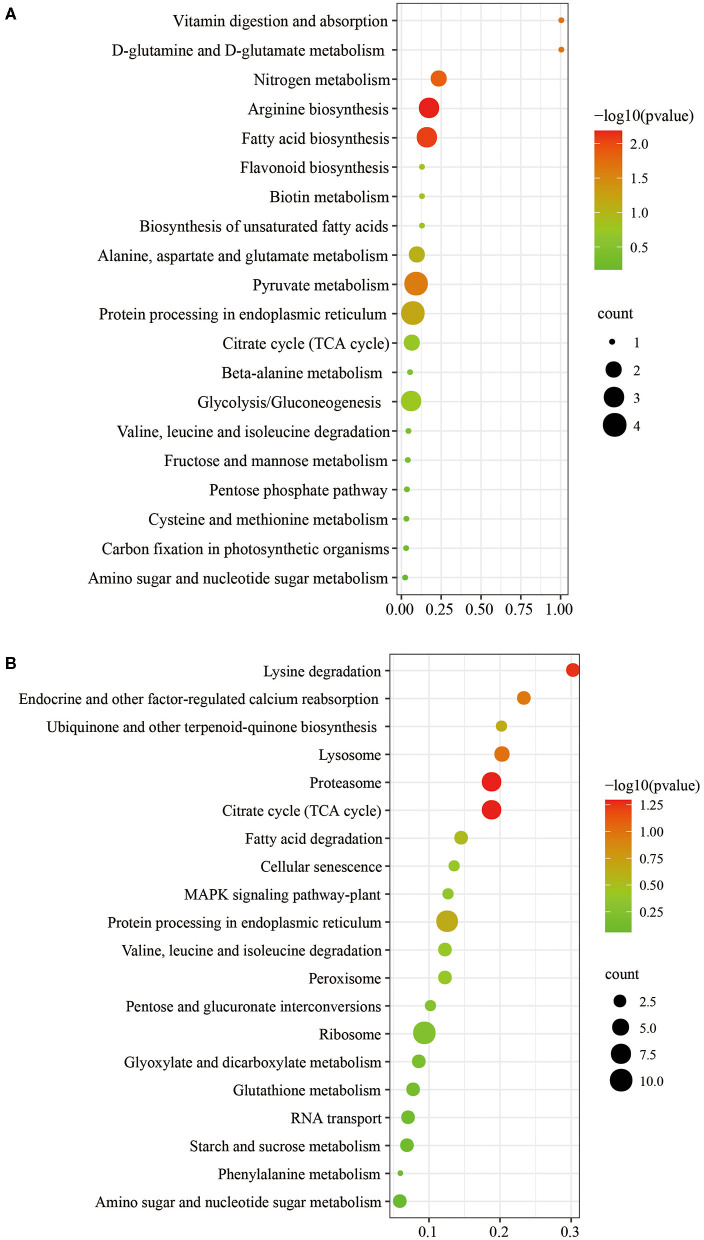
KEGG pathways analysis of DAPs in HR_UR and FR_HR. **(A)** HR_UR; **(B)** FR_HR. The horizontal axis shows the rich factor, which represents the ratio of DAPs to total proteins identified in KEGG pathway functional category. The vertical axis shows the top enriched KEGG pathways. The color and size of each circle indicates *p* values and number of the proteins enriched in each term.

**Figure 6:**
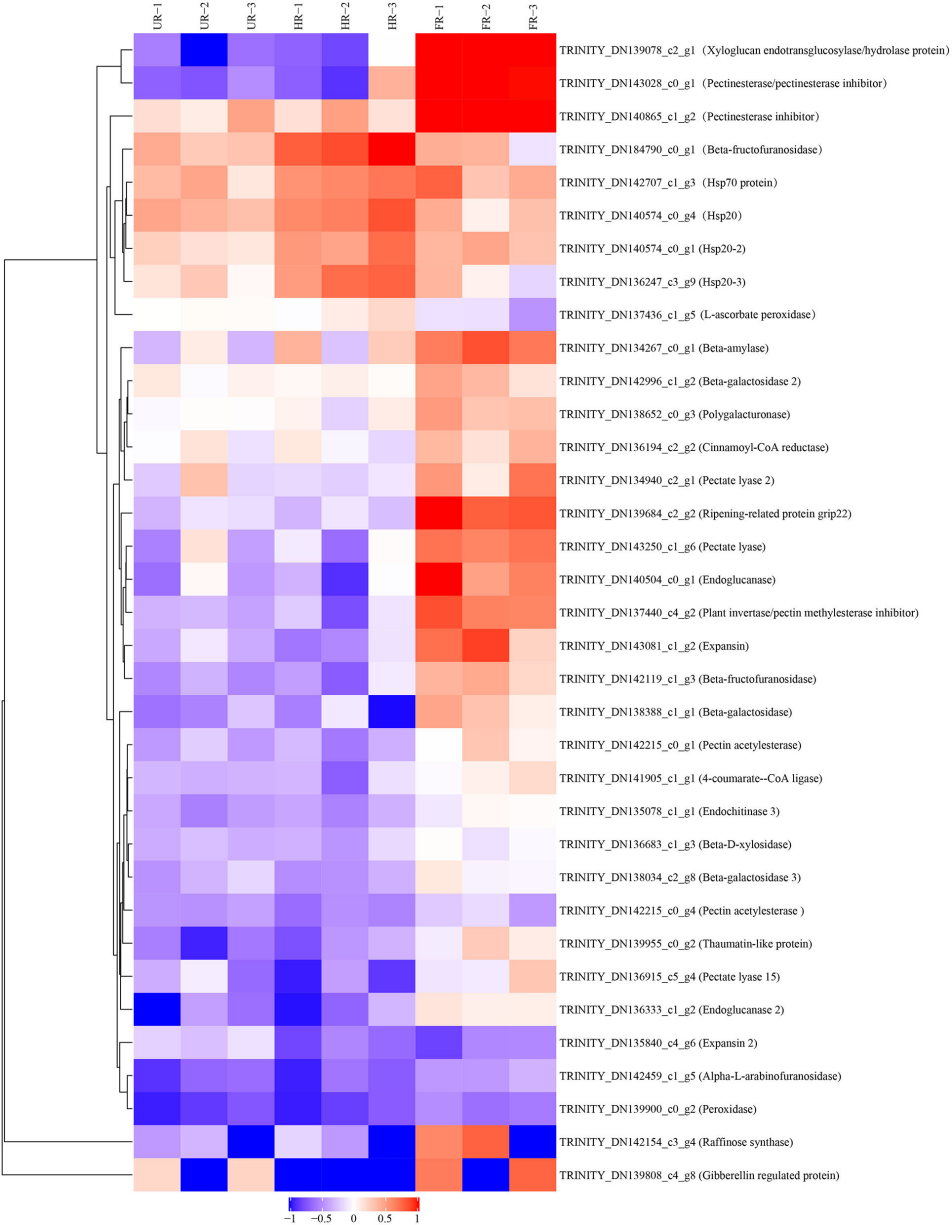
Heatmap analysis of DAPs in UR, HR, and FR (three biological replicates) based on proteomic data, which are associated with cell wall metabolic, plant hormones, and stress response processes.

The biological and functional analysis of these DAPs was performed using GO and KEGG databases to identify processes associated with *A. trifoliata* fruit softening protein targets. GO analysis showed that most of the DAPs were involved in cellular lipid metabolic processes and fatty acid biosynthetic processes in the HR_UR group, whereas biological processes such as macromolecule catabolic processes and carbohydrate transport processes were predominant in the FR_HR group. In terms of molecular function, the main enrichment categories were the oxidoreductase activity and acyl-[acyl-carrier-protein] desaturase activity in the HR_UR group and protein domain-specific binding and copper ion binding in the FR_HR group. Among cell components, the main enrichment categories were plastids in the HR_UR group and cell periphery in the FR_HR group (Figures 4A,B). According to KEGG pathway analyses, pyruvate metabolism, protein processing in the endoplasmic reticulum, and glycolysis/gluconeogenesis were the primarily enriched pathways in the HR_UR group and ribosome, protein processing in the endoplasmic reticulum, and proteasome were the most enriched pathways for DAPs in the FR_HR group (Figures 5A,B). Moreover, protein processing in the endoplasmic reticulum, citrate cycle (TCA cycle), valine, leucine, and isoleucine degradation, and amino sugar and nucleotide sugar metabolism were the enriched pathways in both HR_UR and FR_HR groups. Additionally, previous studies indicated that proteins/genes related to cell wall metabolism, stress and defense responses, and plant hormones might play key roles in fruit ripening and softening (9, 22, 24). Cluster analysis involving cell wall metabolism, stress and defense responses, and plant hormones was therefore performed. The results showed that there were significant differences in protein expression between mature fruit and immature fruit. Furthermore, most of these DAPs were down-regulated in the HR_UR group but up-regulated in FR_HR group (Figure 6).

### Interaction Analysis of DAPs

**Figure 7:**
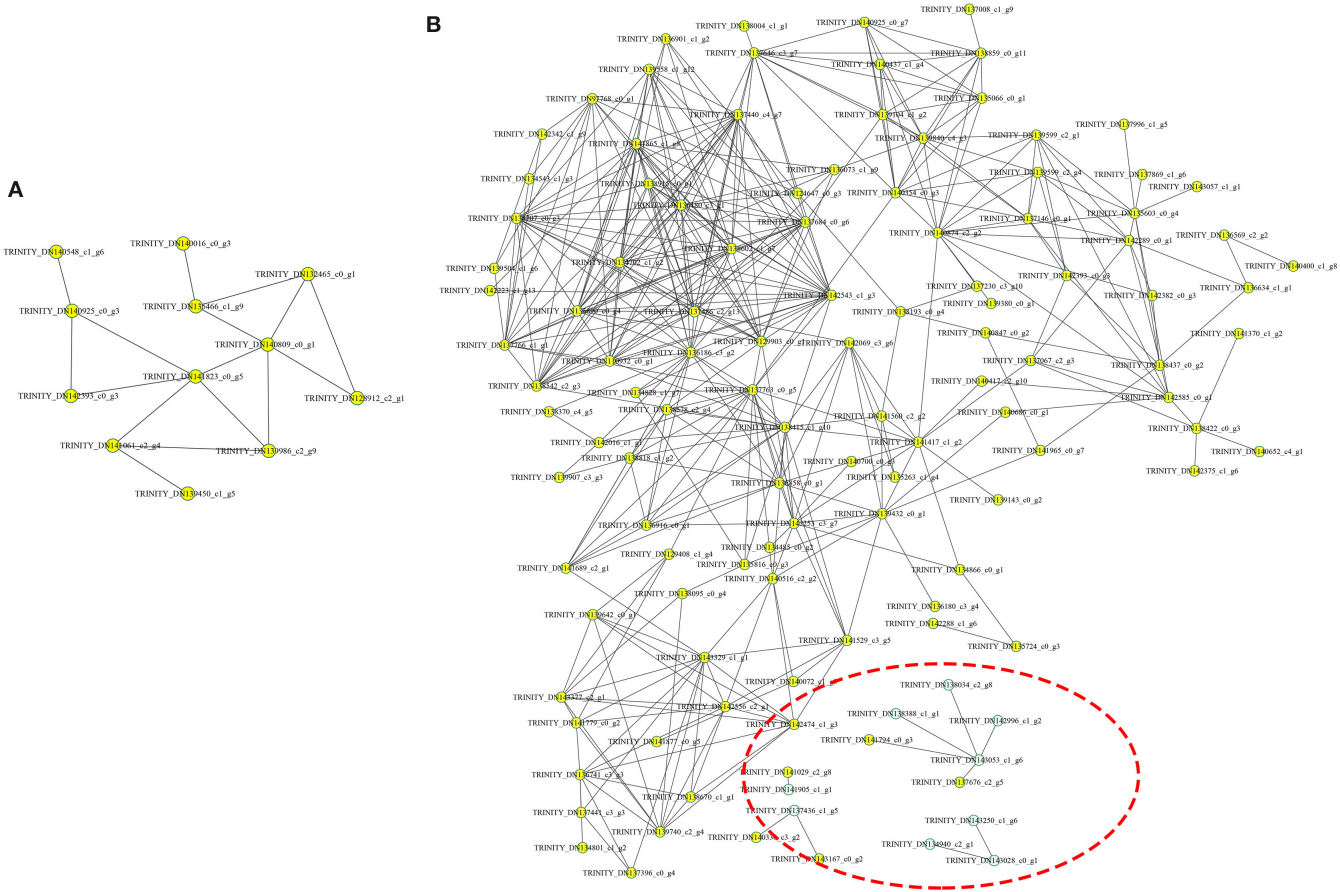
Analysis of the functional network by STRING 9.0 of DAPs. **(A)** The functional network in the HR_UR group. **(B)** The functional network in the FR_HR group. The green circles inside the red dotted line represent proteins that may interact with each other in cell wall and stress response processes.

The protein-protein interaction (PPI) network was analyzed to predict the biological functions during *A. trifoliata* fruit ripening using the STRING database, which indicated that 132 total proteins were assigned to the interaction network (Figure 7). Among these, several proteins involved in cell wall and stress and defense response metabolism, including PE (TRINITY_DN143028_c0_g1), PL1 (TRINITY_ DN143250_c1_g6), PL2 (TRINITY_DN134940_ c2_g1), β-GAL (TRINITY_DN138388_c1_g1), β-GAL2 (TRINITY_DN142996_c1_g2), cysteine protease RD21B (RD21; TRINITY_DN143053_ c1_g6) and β-GAL 3 (TRINITY_DN138034_c2_g8), peroxidase (PRX, TRINITY_DN137436_ c1_g5), 14-3-3 protein (TRINITY_DN140330_c3_g2), 4-coumarate-CoA ligase 2 (4CL; TRINITY_ DN141905_c1_ g1), and UDP-glucoronosyl and UDP-glucosyl transferase (TRINITY_DN141029_ c2_g8), apparently interacted with each other.

### Validation of Data Reliability Through Reverse Transcription Real-Time Quantitative PCR (qPCR)

**Figure 8:**
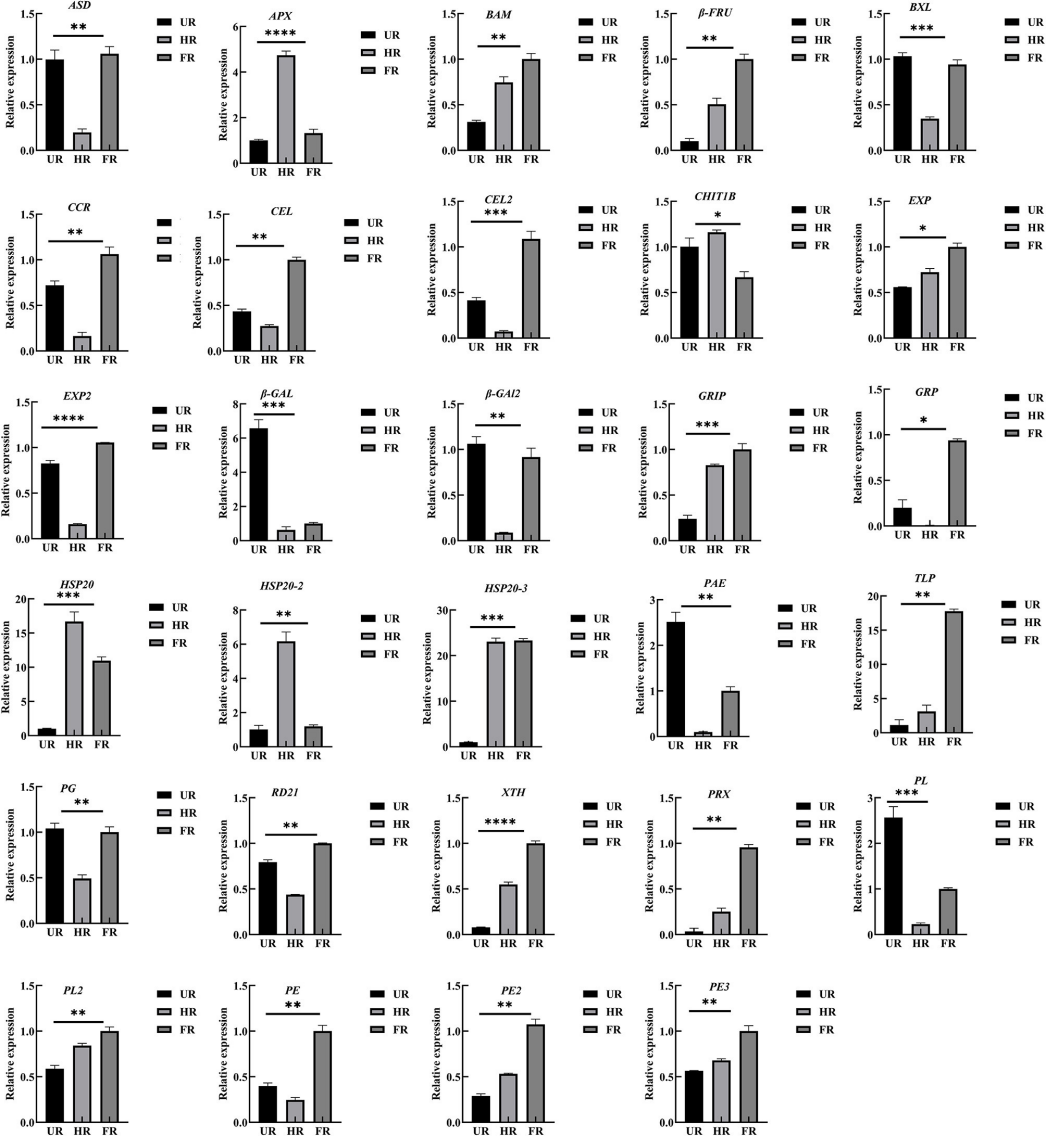
Validation and expression analysis of selected proteins using qPCR. *p < 0.05, **p < 0.01, ***p < 0.001, ****p < 0.0001.

To confirm the proteomic analysis results, 29 DAPs that were mainly associated with cell wall metabolism, plant hormones, and other metabolism were selected for transcription analysis using qPCR. Among these, 13 genes, including *ASD*, β*-GAL1*, β-*GAL2, BXL*, cinnamoyl-CoA reductase (*CCR*), endoglucanase (*CEL1* and *CEL2*), *EXP2*, gibberellin-regulated protein (*RGP*), *PG, PE, PL*, and *RD21* were significantly down-regulated; inactive beta-amylase (*BAM*), *EXP1*, beta-fructofuranosidase (β*-FRU*), *XTH*, ripening-related protein grip22 (*GRIP*), *PE2, PL2, PE3*, pectin acetylesterase (*PAE*), thaumatin-like proteins (*TLP*), heat shock proteins (*HSP20, HSP20-2*, and *HSP20-3*), basic endochitinase (*CHIT1B*), ascorbate peroxidase (*APX*), and peroxidase (*PRX*) were significantly up-regulated in the HR_UR group. Except for *HSP20, HSP20-2, APX*, and *CHIT1B*, the other 25 DAPs were significantly up-regulated in the FR_HR group (Figure 8).

## Discussion

Fruit ripening leads to the development of soft and edible fruit, which is one of the most important changes that determine fruit quality and improve palatability (25). However, only two recent study has reported the use of transcriptome sequencing for *A. trifoliata* fruit (14, 17), and the limited genetic research has impeded an understanding of the genetic basis for its postharvest quality control, consumer acceptance, and breeding for cultivar improvement. Proteomic-based technologies have been extensively applied to analyze many aspects of fruit development; large-scale protein profiling and proteomic analysis provide more direct information on fruit softening at the protein level (9, 26, 27). In this study, a TMT-based comparative proteomics analysis was conducted; 2,839 proteins were identified, and 302 showed variations in abundance in ripe fruit when compared to levels in unripe fruit, which provides a good genetic reference for *Akebia* species.

The functional prediction of proteins could provide information on inner-cell metabolic pathways, as well as the phenotypic and biological implications of genetic variations. GO analysis showed that oxidoreductase activity was enriched in both HR_UR and FR_HR groups, which was consistent with the active oxidoreduction homeostasis that occurs as ripening progresses, suggesting that changes in the antioxidant system are regulated by fruit ripening (28, 29). KEGG analysis indicated that the DAPs were mainly enriched in pyruvate metabolism, arginine biosynthesis, TCA cycle, and protein processing in the HR_UR group, which might lead to good taste and flavor via the accumulation of sugars and total soluble solid content (30). The DAPs in the FR_HR group were enriched in polysaccharide catabolic process and cell wall metabolism, which was associated with changes indicating that cell wall polysaccharides might be involved in fruit softening during ripening (15, 31). Thus, the functional classification of proteins would provide a further understanding of the molecular physiology of fruit ripening and softening. Moreover, the discovery of functional modules in the PPI network plays key roles in the understanding of the organization and function of related biological systems (32). In this study, 132 DAPs were mapped in the PPI network to pathways. Notably, PE (TRINITY_DN143028_c0_g1) and PRX (TRINITY_ DN137436_ c1_g5) were hub nodes in the PPI network and might interact with target proteins to influence fruit ripening and softening in a direct or indirect manner.

Fruit ripening includes significant alterations in many metabolic processes, and many co-expressed proteins are involved in regulating this process. Cell wall metabolism involves the depolymerization of multiple polysaccharide networks through diverse cell wall-modified proteins, leading to fruit softening (33). For example, *PEs, PL, PG*, β*-GAL*, and *PAE* could facilitate its modification and subsequent degradation of pectin polysaccharides, leading to fruit softening (9). *XTHs* and *CEL* are related to the disassembly of the cellulose matrix in the cell wall, contributing to fruit ripening-associated softening (34). The activity level and expression of BXL and ASD are significantly up-regulated in ripened fruit, suggesting that they may be involved in hemicellulose degradation and play important roles in fruit ripening and softening (9, 35). Studies have indicated an increase in *FaEXPA2* expression during strawberry fruit ripening and softening, which is used as molecular marker of strawberry fruit softening (36). In addition, PRXs are important oxidoreductase enzymes and are tightly associated with cell wall remodeling (37). In this study, 11 DAPs involved in cell wall metabolism, namely, *PE, CEL1, PE2, CEL2, PL2, ASD, PRX, EXP1, XTH, PE3*, and *PAE*, showed higher protein and gene expression in ripe flesh than unripe flesh, showing that cell polysaccharides might be very important in fruit ripening and softening (38). Moreover, PL, PE, β-GAL, and PRX were hub modes in the PPI network and might interact with other target proteins to participate in fruit ripening and softening. The significantly increased expression of cell wall-modifying proteins previously indicated a network of cell wall pectin, cellulose, and hemicellulose in fruit ripening and softening (39).

**Figure 9:**
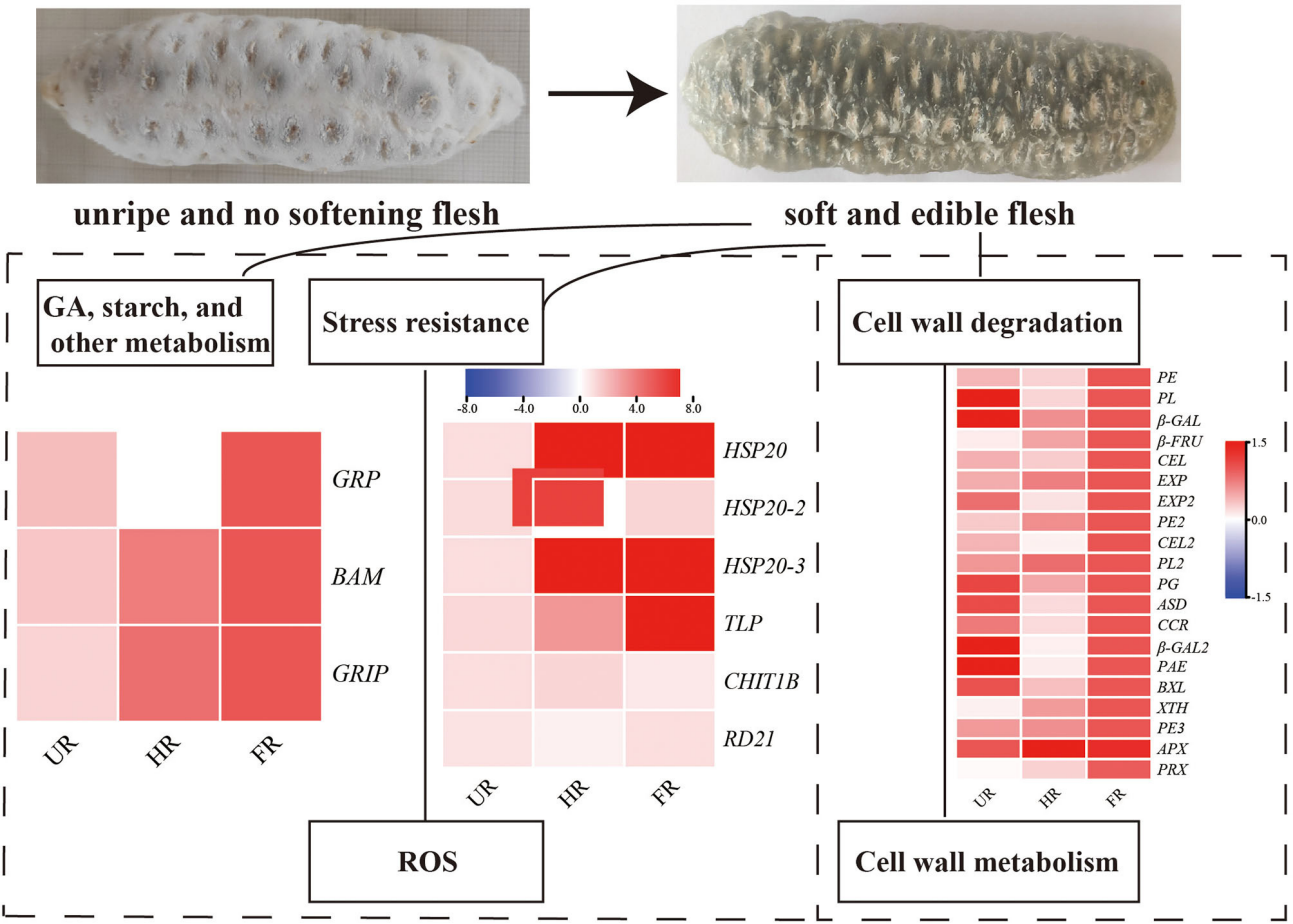
Summary of some of the biological pathways involved in *A. trifoliata* fruit ripening and softening.

The activity of BAM was determined to be increased 10-fold when fruit ripening starts, suggesting that it plays significant roles in fruit maturation and ripening processes, such as starch degradation and fruit softening (40). GRP is involved in the hormonal signaling pathway, and plays crucial roles in plant germination, cell division, flowering, and fruiting. Studies have shown that Snakin/GRP proteins also play important roles in fruit ripening, *Snakin* gene silencing reportedly affects potato cell division and cell wall composition (41). Moreover, β-FRU was found for the first time to be related to hawthorn fruit softening and textural changes (42). The expression of ripening-related protein-GRIP22 is increased during the early ripening of mutant berries and is assumed to be associated with berry fruit softening (43). In this study, the expression of *BAM, GRP*, β*-FRU*, and *GRIP22* was significantly increased in ripened fruit at both mRNA and protein expression levels, indicating that starch, plant hormone, and ripening-related proteins might be associated with starch degradation and fruit softening (Figure 9).

Reactive oxygen species (ROS), including O2^·−^, H_2_O_2_, and ^·^OH, have effects on cell wall disassembly of banana pulp, suggesting that they initiate the disassembly of cell wall polysaccharides and accelerate fruit softening (44). Studies have indicated that the expression of chitinase, endochitinase, cysteine protease, and thaumatin-like proteins are significantly higher in softening fruit than in hard banana fruit, indicating that they may be involved in response to the accumulation of ROS during fruit ripening (9). RNA interference-mediated repression of a cysteine protease gene, *SlVPE3*, in tomatoes delays fruit ripening and increases disease severity (45). Heat shock proteins (HSPs), which are induced by biotic and abiotic stresses, are also expressed during fruit ripening (26). Moreover, as one of the antioxidant enzymes, PRX can improve the ROS-scavenging capacity and decrease the level of ROS in the treated fruit (46). In this study, proteins including *CHIT1B, TLP, RD21*, and *PRX* were up-regulated in the ripened fruit at both mRNA and protein expression levels. *HSP20, HSP20-2*, and *HSP20-3* were up-regulated when the start of fruit ripening at both mRNA and protein expression levels (Figures 6, 8). Notably, β-GAL, β-GAL2 and RD21 may establish functional PPIs, suggesting that the up-regulation of stress resistant-related proteins is important in pathogen resistance and fruit ripening.

In conclusion, 11 DAPs involved in cell wall modification, notably, the proteins in hub modes in the PPI network (PE, PL, β-GAL, and PRX), showed higher expression levels at the ripening stage than at the non-ripening stage at protein and mRNA levels, consistent with results showing that cell wall metabolism might play key roles in fruit ripening and softening (33, 38). Moreover, *BAM, GRP, CHIT1B, TLP, RD21, HSP20, HSP20-2*, and *HSP20-3* also showed high expression levels during fruit ripening. The significantly increased expression of these cell wall metabolism, starch, gibberellin, and stress-responsive proteins, indicating that they might play important roles in cell wall and starch degradation, stress resistance, and *A. trifoliata* fruit ripening and softening. To suppress the expression and activity of cell wall components and retard the commencement of hormone production, 1-methylcyclopropene treatment, and packaging fruit can be performed, which may delay fruit softening (47, 48). Moreover, transgenic plants in which candidate genes are silenced are needed to validate the functions of these genes, and molecular markers for fruit softening will be developed in the future to utilize such traits in breeding and enhance the shelf-life of *A. trifoliata* fruit.

## Data Availability Statement

The datasets presented in this study can be found in online repositories. The names of the repository/repositories and accession number(s) can be found below: https://www.iprox.cn//page/project.html?id=IPX0002000000.

## Author Contributions

JN: designed and performed the study, analyzed the data, and drafted the manuscript. ZS, YS, YZ, and JinC: assisted in analysis and interpreting of data. JinC, ML, KH, and JiaC: acquisition of data and draft preparation. All authors read and approved the final manuscript.

## Conflict of Interest

The authors declare that the research was conducted in the absence of any commercial or financial relationships that could be construed as a potential conflict of interest.

## Supplementary Material

Supplementary Table 1 Sequences of specific primers used for qRT-PCR experiments.

Supplementary Table 2 Summary of differentially accumulated proteins.
